# Nitrate Chemodenitrification by Iron Sulfides to Ammonium under Mild Conditions and Transformation Mechanism

**DOI:** 10.1021/acs.est.4c00195

**Published:** 2024-05-21

**Authors:** Huanhuan Hu, Yang Bai, Chong−wen Zhou, Weihang Jia, Piet N. L. Lens, Zhenhu Hu, David Caffrey, Xinmin Zhan

**Affiliations:** † Civil Engineering, School of Engineering, College of Science and Engineering, University of Galway, Galway H91 TK33, Ireland; ‡ Combustion Chemistry Centre, School of Biological and Chemical Sciences, Ryan Institute, University of Galway, Galway H91 TK33, Ireland; § School of Energy and Power Engineering, Beihang University, Beijing 100191, China; ∥ Department of Microbiology, University of Galway, Galway H91 TK33, Ireland; ⊥ Department of Municipal Engineering, School of Civil Engineering, Hefei University of Technology, Hefei 230009, China; # School of Physics, Trinity College Dublin, Dublin 2, Ireland

**Keywords:** iron sulfides, nitrate chemodenitrification, ammonium, electron release, heterogeneous interface processes, sulfur vacancies

## Abstract

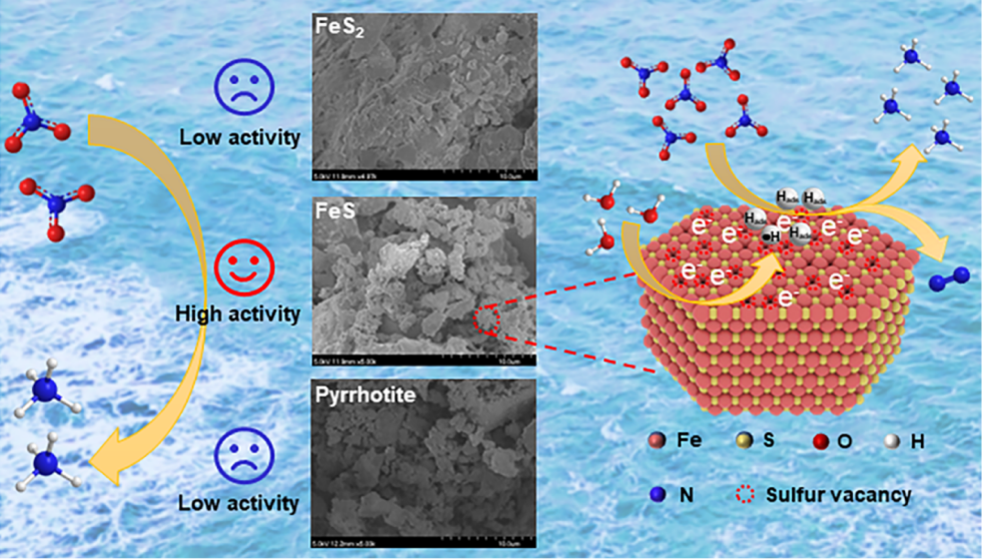

Autotrophic denitrification utilizing iron sulfides as electron donors has been well studied, but the occurrence and mechanism of abiotic nitrate (NO_3_^–^) chemodenitrification by iron sulfides have not yet been thoroughly investigated. In this study, NO_3_^–^ chemodenitrification by three types of iron sulfides (FeS, FeS_2_, and pyrrhotite) at pH 6.37 and ambient temperature of 30 °C was investigated. FeS chemically reduced NO_3_^–^ to ammonium (NH_4_^+^), with a high reduction efficiency of 97.5% and NH_4_^+^ formation selectivity of 82.6%, but FeS_2_ and pyrrhotite did not reduce NO_3_^–^ abiotically. Electrochemical Tafel characterization confirmed that the electron release rate from FeS was higher than that from FeS_2_ and pyrrhotite. Quenching experiments and density functional theory calculations further elucidated the heterogeneous chemodenitrification mechanism of NO_3_^–^ by FeS. Fe(II) on the FeS surface was the primary site for NO_3_^–^ reduction. FeS possessing sulfur vacancies can selectively adsorb oxygen atoms from NO_3_^–^ and water molecules and promote water dissociation to form adsorbed hydrogen, thereby forming NH_4_^+^. Collectively, these findings suggest that the NO_3_^–^ chemodenitrification by iron sulfides cannot be ignored, which has great implications for the nitrogen, sulfur, and iron cycles in soil and water ecosystems.

## Abstract 2

This work provides broader insights into the geochemical field for understanding the cycles of N, Fe, and S in ecosystems and the emergence of life on the Hadean Earth.

## Introduction

In the natural nitrogen cycle, nitrate (NO_3_^–^), nitrite (NO_2_^–^), and ammonium (NH_4_^+^) are the main inorganic nitrogen species in water.^1^ Due to the extensive use of fertilizers in agriculture, along with the discharge of municipal sewage and industrial wastewater, high NO_3_^–^ concentrations in surface and underground water have become a serious environmental issue. This pollution can cause severe environmental damages (i.e., eutrophication) and human diseases (i.e., methemoglobinemia, non-Hodgkin lymphoma, blue baby syndrome, and even cancer).^2,3^ Recently, autotrophic denitrification, conducted by chemolithotrophic denitrifiers using inorganic substances, such as H_2_, elemental sulfur (S), S_2_O_3_^2–^, Fe^0^, Fe^2+^, and iron sulfides, as electron donors has gained intensive attention for efficient NO_3_^–^ removal.^4,5^ Iron sulfides, such as mackinawite (FeS), pyrite (FeS_2_), and pyrrhotite (Fe_1–x_S, x = 0–0.125), as the highly prevalent sulfide minerals in the Earth’s crust and playing an important role in geochemical cycles (nitrogen, phosphorus, sulfur, and iron cycles) in anoxic environments, may represent one of the most promising electron donors for autotrophic denitrification.^5,6^

In the system of iron sulfides-based autotrophic denitrification (IAD), there are a variety of chemical and biochemical reactions involved in NO_3_^–^ reduction, sulfur oxidation, and iron (Fe^2+^) oxidation.^7,8^ Bai et al.’s^7^ research found that the NO_3_^–^ chemodenitrification to NH_4_^+^ took place in an iron sulfide (FeS)-based autotrophic denitrification biofilter under neutral conditions and ambient temperature, which might be described by (eq 1):
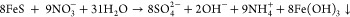
1

However, the chemical reduction process has been ignored or not been observed in many studies on FeS-, pyrrhotite-, and pyrite-based autotrophic denitrification biofilters, in which microbial-driven NO_3_^–^ reduction to N_2_ has been found to be the only NO_3_^–^ reduction process.^8−10^ The generation of a small amount of NH_4_^+^ in IAD is often attributed to the dissimilatory reduction of nitrate to ammonium (DNRA).^11−15^ These conflicting results may be due to the use of different iron sulfides, each with its unique structure and properties, thereby not only influencing the autotrophic denitrification efficiency but also affecting the abiotic chemical reduction of NO_3_^–^ in the IAD systems. Previous studies have emphasized the importance of mineral properties in determining autotrophic denitrification performance.^10^ Therefore, investigating the chemical transformation of NO_3_^–^ with different types of iron sulfides is necessary for an in-depth understanding of potential chemical conversion of NO_3_^–^ by iron sulfides and the cycles of S, Fe, and N in IAD biofilters when chemical and biological NO_3_^–^ transformations take place simultaneously.

Several geochemical studies on the N cycle and early life on earth have observed NO_3_^–^ reduction to NH_4_^+^ by FeS under the conditions of high temperature or acidic to neutral pHs. For instance, Wang et al.,^16^ taking the acid sulfate soil (ASS) environment as the background, explored the N cycle during the NO_3_^–^ chemodenitrification by FeS at ambient temperature (24 °C) and low to neutral pHs (3.5–7). It was found that only 10% of the NO_3_^–^ was reduced to NH_4_^+^ when the pH was 3.5.^16^ Although low pH is common in natural environments, especially in acidic soils, it is important to study the cycling of S, Fe, and N under relatively mild conditions (15–30 °C, pH 6–7.5, and ambient pressure) simulating conditions commonly found in natural ecosystems and engineered wastewater treatment systems, which is beneficial to maintaining biological reactivity without causing adverse effects on microorganisms or enzymatic processes. Additionally, the optimal pH value of the IAD process is recommended at 6.8–8.2.^17^ In some other studies in the field of abiogenesis, NH_4_^+^ production through the chemodenitrification of NO_3_^–^, which was produced in the Hadean oceans by atmospheric reactions possibly driven by electrical discharges or cometary impacts,^18^ by iron sulfides, may exist in and around aqueous environments in the Hadean Eon. NH_4_^+^ was considered a fundamental building block for the formation of amino acids and peptides on Hadean Earth, contributing to the origin of life. These research found that only when the temperature was 120 °C,^19^ or at low to neutral pH value (4.7–6.9) (eq 2),^20^ FeS could reduce NO_3_^–^ to NH_4_^+^ at a low level of 0–6.7% yield:
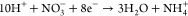
2

While these studies have investigated the effects of various parameters such as pH and temperature on the chemodenitrification of NO_3_^–^ by FeS, the underlying mechanism of this process remains unclear. Wang et al.^16^ detected the presence of reducing sulfur species (such as H_2_S or S_2_O_3_^2–^) and speculated that reducing hydrous sulfides may participate in NO_3_^–^ chemodenitrification or passivation inhibiting electron transfer on FeS surface; however, there was no direct evidence. It is yet to be determined whether this reduction process occurs through a homogeneous ion reaction or a heterogeneous solid–liquid reaction. Therefore, a comprehensive study of the chemical reduction mechanisms of iron sulfides and NO_3_^–^ is not only crucial to explore the transformation mechanism of the N, S, and Fe elements in the IAD system but is also of great significance to comprehend the complexity of the N cycle in ecosystems and even explore the origin of life on the Hadean Earth.

Therefore, the purpose of this study was to explore the performance and mechanism of NO_3_^–^ chemodenitrification by different iron sulfides. The specific objectives of this study were (1) to assess NO_3_^–^ chemical reduction by three common iron sulfides: FeS, ferrous disulfide (FeS_2_), and pyrrhotite; (2) to investigate the chemodenitrification efficiency of NO_3_^–^ with FeS based on the effects of type of nitrates, m(FeS)/m(NO_3_^–^–N) ratio, reaction temperature, and initial pH values; and (3) to explore the mechanism of NO_3_^–^ chemodenitrification by FeS through the identification of reactive species, quenching experiments, and density functional theory (DFT) calculations.

## Materials and Methods

### Materials

FeS (fused sticks, Fe 60–67%, S 25%) and FeS_2_ (extra pure powder, 1.5–4.5 mm) were purchased from Thermo Fisher Scientific (Geel, Belgium). Pyrrhotite (Fe 56.69%, S 38.46%)^4^ was directly obtained from a mine in Tongling City, Anhui Province, China. Before being used in experiments, FeS sticks, FeS_2_ powder, and pyrrhotite were pulverized into fine particles (48–550 μm) using a crusher. NaNO_3_ served as the NO_3_^–^–N source, and all solutions were prepared using ultrapure water (18.2 MΩ cm). The other reagents employed in this study were of analytical grade.

### Experimental Procedures

All experiments were performed in serum glass bottles (160 mL volume) sealed with butyl rubber stoppers. 100 mL of NO_3_^–^–N solution (30 mg/L) was added into the bottles, as well as a given mass (20 g) of the FeS, FeS_2_, and pyrrhotite, respectively. The dosages of iron sulfide and NO_3_^–^ were representative of typical conditions encountered in IAD biofilters.^7,15,21^ After flushing with N_2_ for 15 min, the bottles were sealed and placed in a shaker at 30 °C; this temperature was based on the recommended temperature (28–32 °C) for autotrophic denitrifiers.^17,22^ If not specified, the initial pH (6.37) of the experiments was the unadjusted pH of the solutions because it was relatively neutral and close to the recommended pH value (6.8–8.2) for IAD biofilters and autotrophic denitrifiers,^17,22^ as well as the pH values commonly found in natural ecosystems (pH 6.5–7.5). After reaction for a set time, 2.0 mL samples were extracted from the bottles and immediately filtered through 0.22 μm nylon syringe filters for the measurement of NO_3_^–^–N, NH_4_^+^–N, NO_2_^–^–N, Fe^2+^, and SO_4_^2–^. The effects of type of nitrates, the m(FeS)/m(NO_3_^–^–N) ratio (1667–13333) (the dosage of FeS, 50–400 g/L; NO_3_^–^–N, 30 mg/L) simulating the dosages of iron sulfide materials in IAD biofilters, reaction temperature (20–50 °C), and initial solution pH (2.04–11.95) on NO_3_^–^ reduction were also investigated. The adjustment of the initial solution pH was conducted by introducing either sodium hydroxide (NaOH, 2 M) or sulfuric acid (H_2_SO_4_, 2 M). Although some temperatures and pH values were assessed not common in the natural environment or wastewater treatment systems, the study of these temperatures and pH values would help to elucidate the mass transfer, activation, and adsorption mechanisms of NO_3_^–^ chemodenitrification by FeS. All experiments were conducted in duplicate.

### Analytical Methods

The comprehensive analysis of this study, including the concentrations of NO_3_^–^–N, NH_4_^+^–N, NO_2_^–^–N, Fe^2+^, S_2_O_3_^2–^, SO_3_^2–^, and SO_4_^2–^, is all displayed in Text S1. Additionally, the characterization methods on Tafel scans, X-ray fluorescence (XRF), X-ray diffraction (XRD), X-ray photoelectron spectroscopy (XPS), field emission scanning electron microscopy (FESEM), and Zetasizer are also shown in Text S1.

## Results and Discussion

### Reduction of NO_3_^–^ by Iron Sulfides

**Figure 1:**
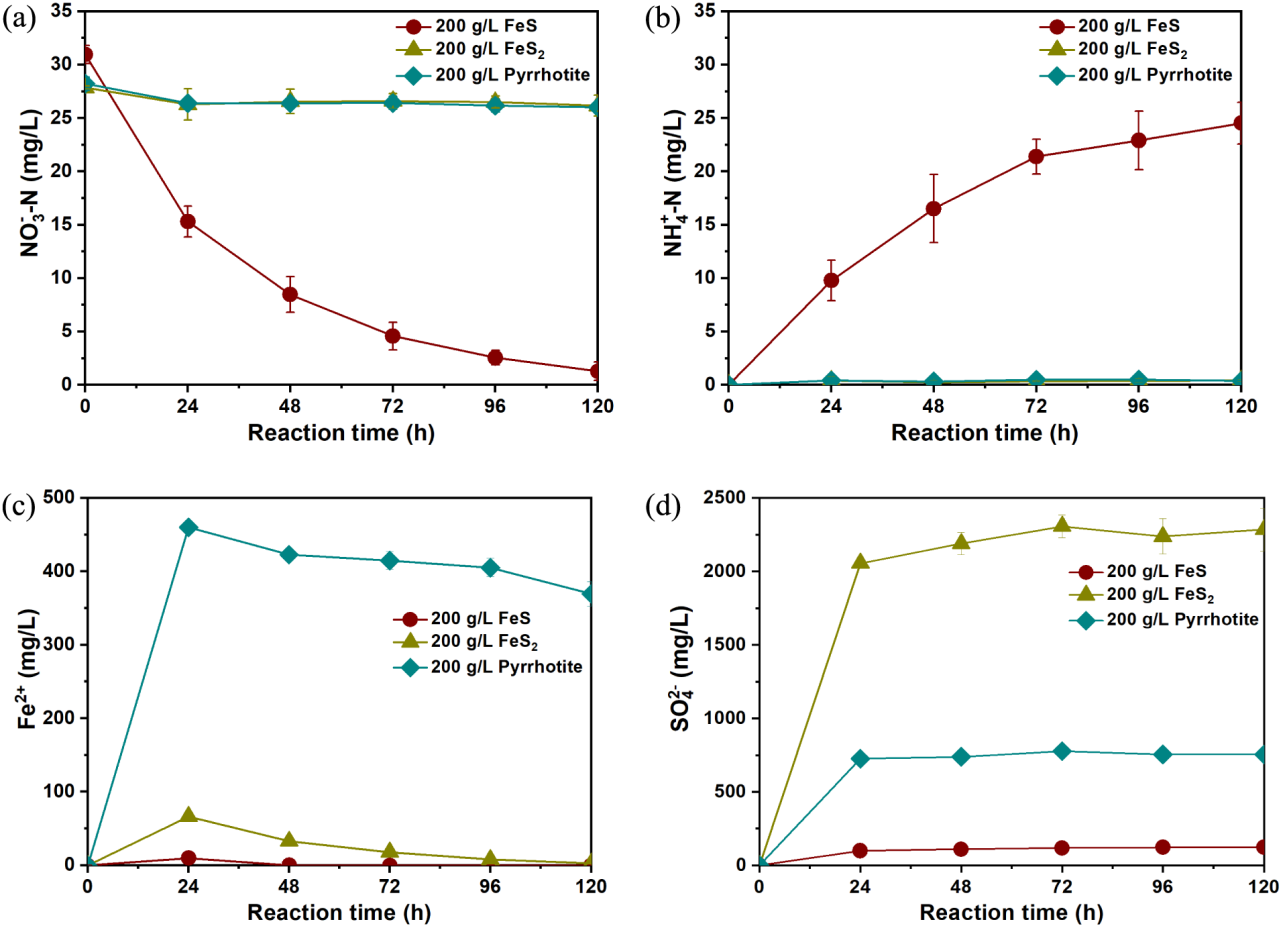
Reduction of NO_3_^–^ by iron sulfides: variation of (a) NO_3_^–^–N, (b) NH_4_^+^–N, (c) aqueous Fe^2+^, and (d) aqueous SO_4_^2–^. The error bars represent the standard deviations calculated from three parallel samples. Experimental conditions: [iron sulfide]_0_ = 200 g/L, [NO_3_^–^–N]_0_ = 30 mg/L, and *T* = 30 °C, without pH adjustment.

Figure 1a presents NO_3_^–^ chemodenitrification by iron sulfides under anoxic conditions. FeS chemically reduced NO_3_^–^ at 30 °C and unadjusted initial pH, and the NO_3_^–^–N reduction efficiency was 97.5% ± 0.1% after 120 h, while the NO_3_^–^ chemodenitrification was almost negligible within 120 h when FeS_2_ and pyrrhotite were the reactants. It was observed that the main NO_3_^–^ chemodenitrification product by FeS was NH_4_^+^, and the concentration of NH_4_^+^–N increased to 24.53 (±1.95) mg/L at 120 h. NO_2_^–^ was not detected during the reduction of NO_3_^–^ by FeS (Figure S1). However, the amount of NH_4_^+^–N produced (24.53 ± 1.95 mg/L) did not account for the total NO_3_^–^–N removed (29.70 ± 0.81 mg/L) from solution by FeS (Figure 1a,b); the other product of NO_3_^–^ reduction was N_2_ with the concentrations of NO, NO_2_, and N_2_O in the headspace gas phase below the limit of detection. Many previous studies observed N_2_O emission during the chemodenitrification process of Fe(II) and NO_3_^–^/NO_2_^–^.^23−27^ However, in the present study, negligible N_2_O was produced. Although conditions of this study (30 °C, pH 6.37) were largely similar to the previous studies (5–35 °C, pH 5.5–7.0)^25,27^ and all involved Fe(II), previous studies were mainly focused on NO_2_^–^ chemodenitrification or NO_3_^–^ chemodenitrification in the presence of buffer media and catalysts,^28^ while in this study, no any buffer media and catalysts were added and the production of NO_2_^–^ was not detected. In Kalina’s research,^26^ the structure of iron (Fe-rich smectite clay mineral nontronite and the mixed Fe(II)–Fe(III) oxyhydroxide phase green rust) played a crucial role in the process of chemodenitrification of NO_2_^–^ to produce N_2_O,^26^ but this iron(II) structure was not detected in the XRD pattern of FeS samples before and after NO_3_^–^ chemodenitrification in this study. Therefore, different NO_3_^–^ chemodenitrification pathways might have resulted in different N_2_O emissions.

During the NO_3_^–^ removal process, the concentrations of aqueous Fe^2+^ in the FeS system were about 9.74 mg/L at 24 h and then consistently remained below 0.1 mg/L, and dissolved Fe^3+^ was not detectable. These results could be attributed to the pH of the solution increasing from 6.37 to 9.78 in the FeS system during the NO_3_^–^ reduction, leading to the precipitation of iron ions. The concentrations of Fe^2+^ in the FeS_2_ system were from 66.60 mg/L at 24 h to 2.94 mg/L at 120 h and were consistently around 400 mg/L in the pyrrhotite system (Figure 1c). In comparison to the FeS system, higher concentrations of Fe^2+^ were observed in both the FeS_2_ and the pyrrhotite systems. This discrepancy may arise from the delayed formation of iron ion precipitation, likely attributable to the pH levels of the FeS_2_–NO_3_^–^ system (5.67) and the pyrrhotite–NO_3_^–^ system (5.97) after 120 h. Alternatively, these differences could be linked to the unique characteristics and reactivity inherent to FeS_2_ and pyrrhotite. FeS_2_ has a cubic crystal structure, and the presence of sulfur–sulfur bonds within the crystal lattice contributes to its stability.^29^ The crystal structure of pyrrhotite is less ordered and may contain vacancies or substitutions.^30^ This structural variability could increase its reactivity compared to FeS_2_ potentially leading to a significant dissolution of Fe^2+^. However, no chemical reduction of NO_3_^–^ occurred in these two systems, indicating that Fe^2+^released from iron sulfides in the aqueous phase cannot chemically reduce NO_3_^–^. The solution after NO_3_^–^ chemodenitrification by FeS was alkaline mainly because FeS is dissolved in a non-oxidizing manner, releasing iron ions into the solution, and the resulting sulfide ions consume protons to form HS^–^ or H_2_S (depending on the pH value).^31^ In addition, as shown in eq 1, the formation of NH_4_^+^ consumes protons and consequently causes pH to rise. Although the alkaline environment was the result of NO_3_^–^ chemodenitrification by FeS, it was not the decisive factor in promoting NO_3_^–^ chemodenitrification by FeS. When the initial pH was acidic, as discussed in the next section, NO_3_^–^ chemodenitrification by FeS still took place.

In addition, the concentrations of SO_4_^2–^ increased in all three systems (Figure 1d). This may be due to the dissolution of SO_4_^2–^ on the surface of the oxidized iron sulfides into the water or the product SO_4_^2–^ formed after the reaction of the sulfur in the iron sulfides with NO_3_^–^. However, for the FeS system with NO_3_^–^ reduction occurring, the measured concentrations of SO_4_^2–^ (122.85 ± 6.71 mg/L) were lower than the stoichiometric value (168.53 mg/L) of SO_4_^2–^ calculated according to stoichiometric eqs 1 and 3(7) in consideration that 2.21 mM of NO_3_^–^–N was reduced to 1.75 mM of NH_4_^+^–N and 0.18 mM N_2_. Furthermore, the concentrations of S_2_O_3_^2–^ and SO_3_^2–^ in the solution detected by IC were negligible. These results indicate that incomplete sulfur oxidation or electron transfer occurred on the surface of FeS under this circumstance.
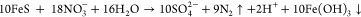
3

### Impacts of Conditions on NO_3_^–^ Chemodenitrification

The type of nitrates or cations affects the solution properties such as ionic strength and consequently might affect the efficiency and selectivity of chemodenitrification of NO_3_^–^. Additionally, reaction parameters, such as the mass ratio of FeS/N, reaction temperature, and initial solution pH, might affect the mass transfer, activation, and adsorption during the removal of NO_3_^–^ by FeS. Therefore, it is imperative to examine their impacts on the NO_3_^–^ chemodenitrification efficacy in the FeS system.

**Figure 2:**
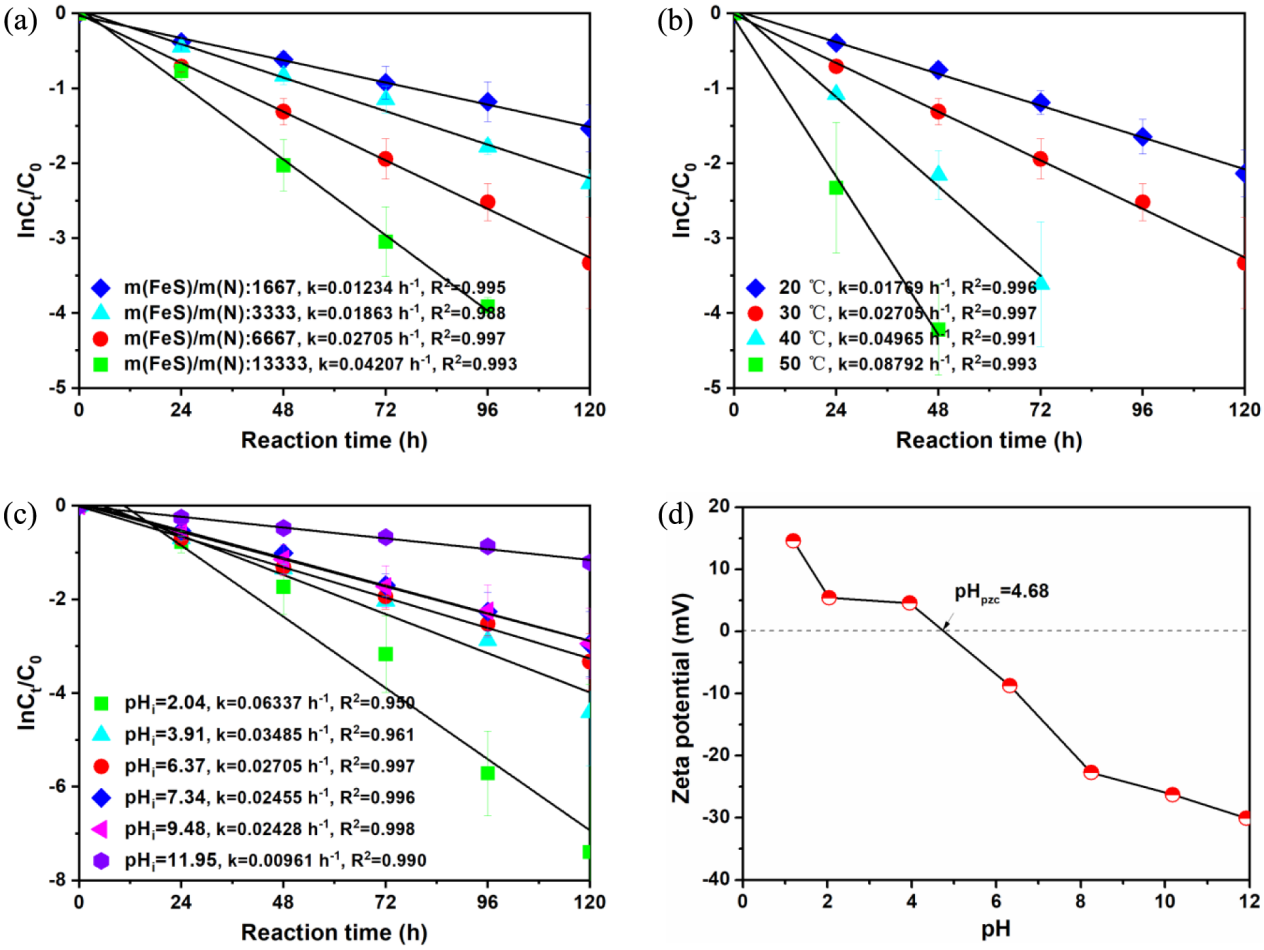
Effects of reaction conditions on NO_3_^–^–N removal for FeS: (a) Mass ratio of FeS/N, (b) temperature, and (c) pH value. Except for the investigated parameters, other parameters were fixed: [FeS]_0_ = 200 g/L, [NO_3_^–^–N]_0_ = 30 mg/L, and *T* = 30 °C, without pH adjustment. (d) Zeta potential analysis of FeS in relation to the pH of the solution.

It can be seen from Figures 1a and S4 that when three types of NO_3_^–^ (NaNO_3_, KNO_3_, and NH_4_NO_3_) were used, the chemodenitrification efficiency of NO_3_^–^ by FeS_2_ and pyrrhotite was almost negligible, but only occurred by FeS. The chemodenitrification efficiency of KNO_3_, NH_4_NO_3_, and NaNO_3_ by FeS (with or without Ca^2+^) and the selectivity of NH_4_^+^ production did not change greatly. These results indicate that the type of nitrate and the cations would not significantly impact the NO_3_^–^ chemodenitrification. In Figure 2a, it is evident that the NO_3_^–^ chemodenitrification efficacy was obviously improved with increasing the mass ratio of FeS/N from 1667 to 13 333 because more FeS can provide more active sites. The selectivity of NH_4_^+^ did not change significantly (Figure S2a). Similarly, raising the reaction temperature also accelerated the reduction of NO_3_^–^ (Figure 2b). This was likely because of the accelerated molecular movement accompanying the temperature increase, thereby promoting mass transfer in the heterogeneous system. Through the plots of ln k and 1/T according to the Arrhenius equation (eq S2) (Figure S3), the activation energy (Ea) for NO_3_^–^–N removal was determined to be 36.78 kJ mol^–1^. This value exceeds the activation energy typically associated with the diffusion-controlled processes (<20 kJ mol^–1^) and lower than chemical reaction steps (>80 kJ mol^–1^), such as bond breaking, indicating that the NO_3_^–^ chemodenitrification process was dominated by heterogeneous reactions occurring on the FeS surface.^32^ Furthermore, as the reaction temperature was elevated from 20 to 50 °C, the selectivity of the product NH_4_^+^ increased correspondingly (Figure S2b). This could be attributed to the higher temperature leading to a greater number of NO_3_^–^ molecule bound to the surface of FeS, thereby facilitating the surface-mediated activation of NO_3_^–^ adsorption and NH_4_^+^ generation.^19^

As illustrated in Figure 2c, the removal efficiency of NO_3_^–^–N gradually declined from 100% to 70% as the initial pH increased from 2.04 to 11.95. These observations could be clarified by considering the consumption of proton (H^+^) ions during the NO_3_^–^ removal reaction, as shown in eq 2.^20^ Furthermore, the solution pH has the potential to modify the electrical properties of the FeS surface, influencing its electrostatic interaction (adsorption or repulsion) with NO_3_^–^.^33^Figure 2d displays that the zeta potential values of FeS declined with increasing pH values. When the pH was below pH_zpc_ (pH at the zero point of charge), the positively charged FeS surface could electrostatically attract negatively charged NO_3_^–^ species, facilitating the reductive conversion of NO_3_^–^. However, an increase in pH would lead to electrostatic exclusion between negatively charged FeS and anionic NO_3_^–^ species, thereby not favoring the reduction efficiency. Figure 2c shows that the reaction rate constants at pH 6.37, 7.34, and 9.48 exhibited slight differences, despite the fact that the proton concentration increased significantly. This could be attributed to the negatively charged surface of FeS when the solution pHs were 6.37, 7.34, and 9.48 (Figure 2d), so there might exist an effect of electrostatic repulsion with the reactant NO_3_^–^, which resulted in no significant increase in the reaction rate. In addition, even though an increase in the proton concentration might drive the equilibrium toward the side of the reaction that consumed protons, the equilibrium might not shift significantly due to factors like Le Chatelier’s principle, which predicts that a system will adjust to counteract changes in conditions (like pH). As observed in Figure S2c, the selectivity of the NH_4_^+^ product during the NO_3_^–^ reduction increased obviously at an initial pH 2.0. This increase could be attributed to the existence of additional positive charges on the surface of FeS (as shown in Figure 2d) at pH 2.04, which could attract more electrostatically negative NO_3_^–^ species. Additionally, the increased concentration of H^+^ in the solution, with H^+^ actively participating in the reaction through the formation of H_ads_ on the FeS surface, increased the probability of a collision with N intermediates. This resulted in a more readily reduction to NH_4_^+^.^34^ It is noted that some experimental groups with high reaction rates displayed large standard deviations. NO_3_^–^ chemodenitrification by FeS was a heterogeneous reaction, so slight differences in the distribution or activity of reaction sites in the parallel experiments would cause observed differences in reaction rates.

### Characterization of Iron Sulfides

SEM imaging (Figure S5) shows that FeS, FeS_2_, and pyrrhotite pulverized by the crusher were all made of monomer particles and tiny particles. The chemical NO_3_^–^ reduction by FeS was not due to its particle morphology being different from that of FeS_2_ and pyrrhotite. XRF (Table S1) analysis shows the main element contents in iron sulfides that had not been exposed to NO_3_^–^, included 27.60% S and 71.50% Fe in FeS; 39.90% S and 45.40% Fe in FeS_2_; 34.90% S and 62.60% Fe in pyrrhotite; and other impurities such as Si, Ca, Mg, and Al. The XRF results show that the mass ratios of sulfur and iron in FeS were 27.60% and 71.50%, respectively, which were inconsistent with the theoretical mass ratios of the molecular formula of FeS (sulfur: 36.36% and iron: 63.64%), indicating that excess Fe existed in FeS, compared to S. So, the FeS contained iron oxides or impurity iron power. Based on the actual measured mass content of the sulfur element (27.60%) in the FeS used in this study, it is calculated that the theoretical mass content of the element iron in FeS amounted to 48.3%. This implies that there may exist at maximum 23.2% impurity iron powder in FeS samples, indicating that the maximum amount of impurity iron powder contained in the system of the NO_3_^–^ reduction by 200 g/L FeS was 46 g/L. In order to explore whether 46 g/L of iron powder could contribute to NO_3_^–^ chemodenitrification, the experiment on NO_3_^–^ chemodenitrification by a mixture of 154 g/L FeS and 46 g/L iron powder, only 46 g/L iron powder, 154 g/L FeS, or 200 g/L FeS were conducted. As shown in Figure S6, the efficiency of NO_3_^–^ chemodenitrification by 46 g/L iron powder alone was 22.7% ± 7.5%; The efficiency of NO_3_^–^ chemodenitrification by the mixture of 154 g/L FeS and 46 g/L iron powder (89.4% ± 2.0%) was not significantly improved compared to by 154 g/L FeS alone (88.6% ± 2.5%), and was lower than that of by 200 g/L FeS (97.5% ± 0.1%). Therefore, it can be inferred that the performance of NO_3_^–^ chemodenitrification by FeS was not attributed to the possible presence of iron powder in the FeS and that the NO_3_^–^ chemodenitrification efficiency was not significantly improved when iron powder and FeS coexisted.

**Figure 3:**
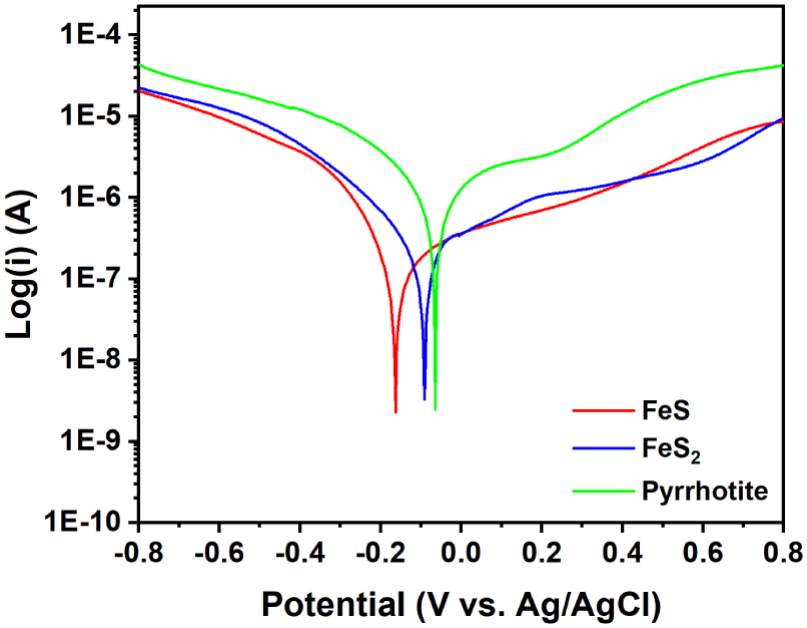
Tafel scans of FeS, FeS_2_, and pyrrhotite in 30 mg/L of NO_3_^–^–N solution.

To unravel the reasons for the superior NO_3_^–^ removal performance by FeS, Tafel scans were first used to check the electron release ability of FeS, FeS_2_, and pyrrhotite in solutions from thermodynamic point of view by measuring the free corrosion potential,^35^ as the more negative value of the free corrosion potential reflects that the material is more likely to lose electrons.^36^ The free corrosion potentials of FeS, FeS_2_, and pyrrhotite amounted to −0.16, –0.09, and −0.06 V, respectively (Figure 3), suggesting that the electron release from FeS was easier than that from FeS_2_ and pyrrhotite.

**Figure 4:**
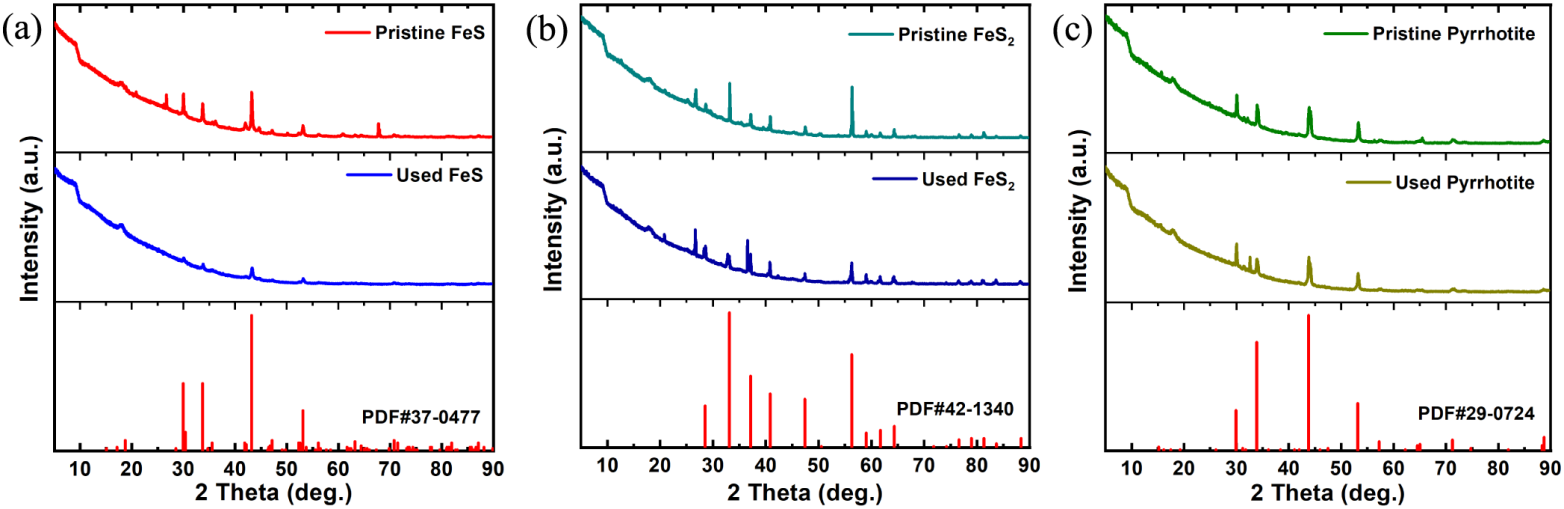
XRD patterns of (a) FeS, (b) FeS_2_, and (c) pyrrhotite samples before (pristine) and after (used) the reaction compared with the standard materials.

XRD spectra clearly indicate that FeS, FeS_2_, and pyrrhotite were highly pure crystal structures (Figure 4). For FeS, the characteristic diffraction peaks at 2θ of 29.92, 33.67, 43.15, and 53.12 Å were assigned to the phases (110), (112), (114), and (300) for troilite–2H (FeS), respectively (PDF no. 37–0477). The characteristic diffraction peaks at 2θ of 36.04, 41.93, and 60.76 Å were assigned to the phases (111), (200), and (220) for wüstite and syn (FeO), respectively (PDF no. 06–0615). Reacted solids after exposure to NO_3_^–^ showed a significant reduction in the characteristic peak of troilite–2H (FeS) (Figure 4a). No characteristic peaks of iron powder were found in the XRD characterization results of FeS before and after the reaction, which also indicates that there was no elemental iron or the trace amount of elemental iron was below the limit of detection in FeS. For FeS_2_, the diffraction peaks observed at 2θ values of 28.51, 33.08, 37.11, 40.78, 47.41, and 56.28 Å were attributed to the (111), (200), (210), (211), (220), and (311) crystal planes of pyrite (FeS_2_), respectively (PDF no. 42–1340).^37^ For pyrrhotite, the peaks at 2θ of 29.92, 33.84, 43.76, and 53.11 Å were in accordance with (200), (205), (2010), and (220) crystal planes of pyrrhotite–5T (Fe_1–x_S), respectively (PDF no. 29–0724).^37^ The characteristic diffraction peaks at 2θ of 44.03 and 71.55 Å were assigned to the phases (206) and (406) for pyrrhotite–3T and syn (Fe_7_S_8_), respectively (PDF no. 24–0220), and the characteristic diffraction peak at 2θ of 65.44 Å was assigned to the phases (800) for pyrrhotite–4 M (Fe_7_S_8_) (PDF no. 29–0723). After exposure to NO_3_^–^, the characteristic peaks of FeS_2_ and pyrrhotite solids did not change significantly compared to those of FeS (Figure 4). The differences in the XRD spectra of the three iron sulfides confirmed the chemical reduction reaction between NO_3_^–^ and FeS.

**Figure 5:**
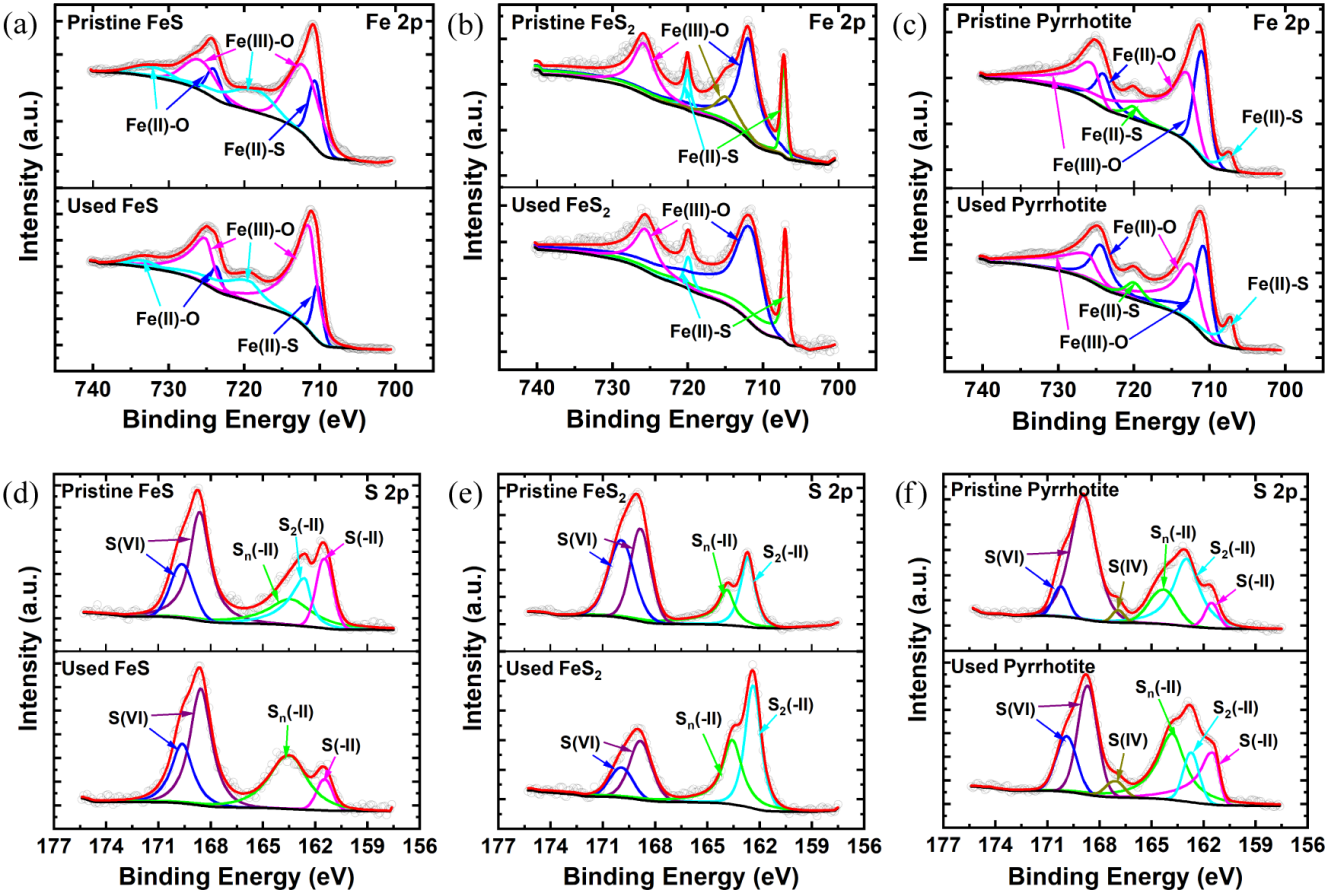
XPS spectra of the FeS, FeS_2_, and pyrrhotite before (pristine) and after (used) the reaction: (a)–(c) Fe 2p and (d)–(f) S 2p.

XPS was used to analyze the changes in surface compounds and electronic states of FeS, FeS_2_, and pyrrhotite before and after NO_3_^–^ reduction. As depicted in Figure 5a, the XPS spectra of Fe 2p peaks of FeS at 710.61, 712.16/718.20/725.85, and 724.08/731.99 eV were identified as corresponding to Fe(II)–S, Fe(III)–O, and Fe(II)–O, respectively, indicating the partial oxidation of Fe(II) on the FeS surface.^38^ After the NO_3_^–^ reduction reaction, the percentage of Fe(II) species (Fe(II)–S and Fe(II)–O) decreased from 27.88% to 14.69%, indicating the oxidation of Fe(II) into Fe(III) species by NO_3_^–^ on the FeS surface. In contrast, the Fe(II) species on the FeS_2_ and pyrrhotite surface did not decrease significantly before and after the reaction (Table S2 and Figure 5b,c). Furthermore, for the S species on the FeS surface, S(−II), S_2_(−II), and S_n_(−II) (*n* = 3, 4, ...) comprised approximately 49.64% of the total S in the fresh FeS sample (Figure 5d); after the reaction with NO_3_^–^, the disappearance of S_2_(−II) (16.43%) in the S 2p and the decrease of S(−II) (from 15.03% to 7.84%), together with the increase in the proportion of S_n_(−II) (from 18.18% to 32.94%) and S(VI) (from 50.35% to 59.22%), indicate that these S species might serve as the reductants for NO_3_^–^ removal and/or the electron donor to promote the Fe(II)/Fe(III) cycle. In contrast, for FeS_2_ and pyrrhotite, the concentrations of S(VI) decreased significantly (Table S2 and Figure 5e,f). Considering the obvious increase of the SO_4_^2–^ concentrations in solutions (Figure 1d), inherent S(VI) might be detached from the surface of FeS_2_ and pyrrhotite during the reaction. To conclude, both XRD and XPS results confirm that FeS participated in the chemical reduction of NO_3_^–^, while FeS_2_ and pyrrhotite did not.

### Role of Aqueous Fe and S Species in the Process of NO_3_^–^ Chemodenitrification on FeS

**Figure 6:**
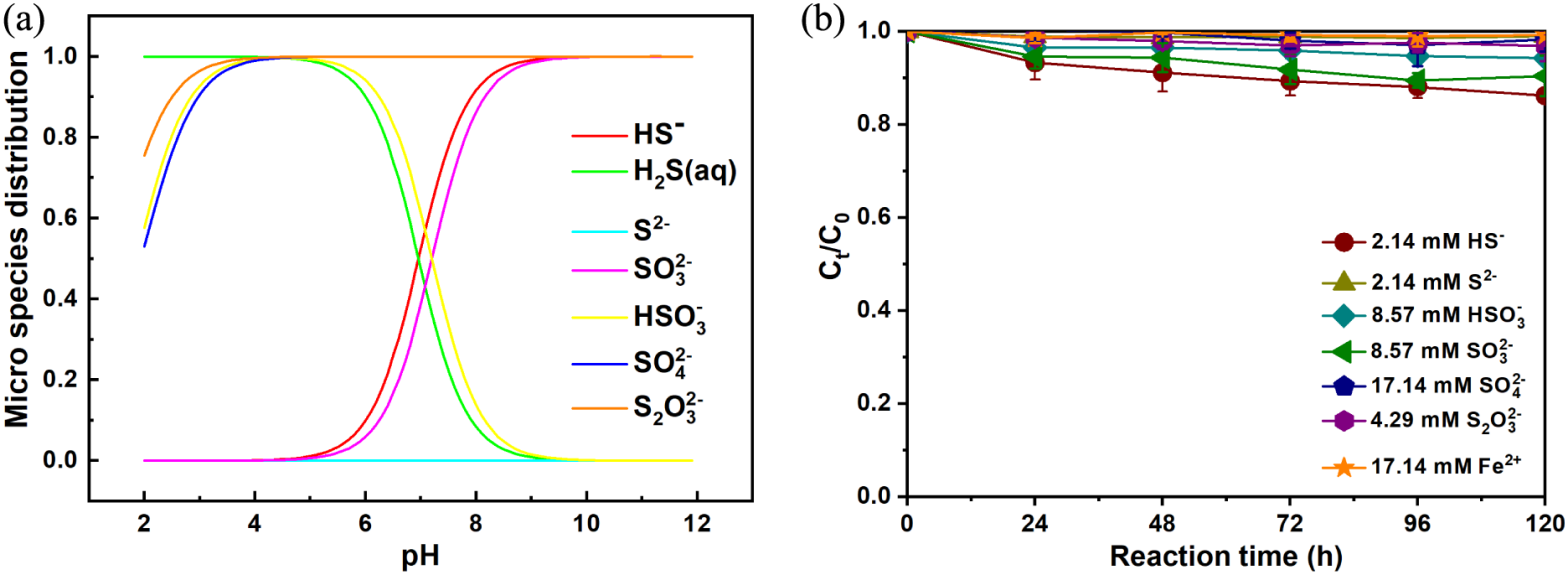
(a) Speciation of sulfur species calculated with Visual MINTEQ 3.1 and (b) reduction of NO_3_^–^ by aqueous sulfur and Fe^2+^ species. Experimental conditions: [NO_3_^–^–N]_0_ = 30 mg/L and *T* = 30 °C, without pH adjustment.

Both Fe(II) present on the FeS surface and Fe^2+^ released from the dissolution of FeS can serve as the active species for NO_3_^–^ chemodenitrification. However, as shown in Figure 6b, soluble Fe^2+^ did not have the ability to reduce the level of NO_3_^–^ (30 °C, without pH adjustment). In addition, as shown in Figure 1c, the concentrations of aqueous Fe^2+^ were quite low. Moreover, the XPS spectra for the Fe 2p regions of pristine and used FeS show that the proportion of Fe(II) obviously decreased during the reduction of NO_3_^–^. Therefore, soluble Fe^2+^ was not the main cause for the chemical reduction of NO_3_^–^.

As the S^2–^ released from FeS undergoes hydrolysis and electron donation processes, it might be transformed into various sulfur species such as HSO_3_^–^, HS^–^, H_2_S, S_2_O_3_^2–^, and SO_3_^2–^.^39,40^ The reduction of NO_3_^–^ by some reducing sulfur species, such as H_2_S, is thermodynamically favorable over a wide pH range,^41^ so the contributions of sulfur species in the NO_3_^–^ reduction process need to be considered. According to the simulation results of Visual MINTEQ 3.1 (the simulation procedure is detailed in Text S1), the distribution of sulfur species changes with pH, as shown in Figure 6a. As the solution pH was increased from initial 6.37 to 9.05–10.73 during the reaction, aqueous H_2_S, HSO_3_^–^, HS^–^, S_2_O_3_^2–^, SO_3_^2–^, and SO_4_^2–^ were the dominant sulfur species. However, the reduction of NO_3_^–^ in all HS^–^, S_2_O_3_^2–^, SO_3_^2–^, SO_4_^2–^, and HSO_3_^–^ systems was almost negligible (Figure 6b). Therefore, it is speculated that the sulfur species in the aqueous phase would not be the main active species for NO_3_^–^ reduction.

### NO_3_^–^ Chemodenitrification Pathway by FeS

From the above analysis, it is quite clear that the reducing ions (Fe^2+^, HS^–^, HSO_3_^–^, SO_3_^2–^, and S_2_O_3_^2–^) in the solution were not the main active species for reducing NO_3_^–^. Therefore, NO_3_^–^ reduction was dominated by the interface processes on the FeS particle surface rather than the soluble active species leached from the material. The XPS peak showed the atomic ratios of S:Fe were 0.54, 1.93, and 1.06 in FeS, FeS_2_, and pyrrhotite, respectively. This indicates that high concentrations of sulfur vacancies (Vs) existed on FeS. At the sulfur vacancies sites, the back-donation of localized electrons would enhance the heterolytic dissociation of adsorbed H_2_O molecules, leading to the generation of •H or (e^–^ and H^+^) (eqs 4 and 5):^42^
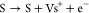
4
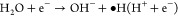
5

**Figure 7:**
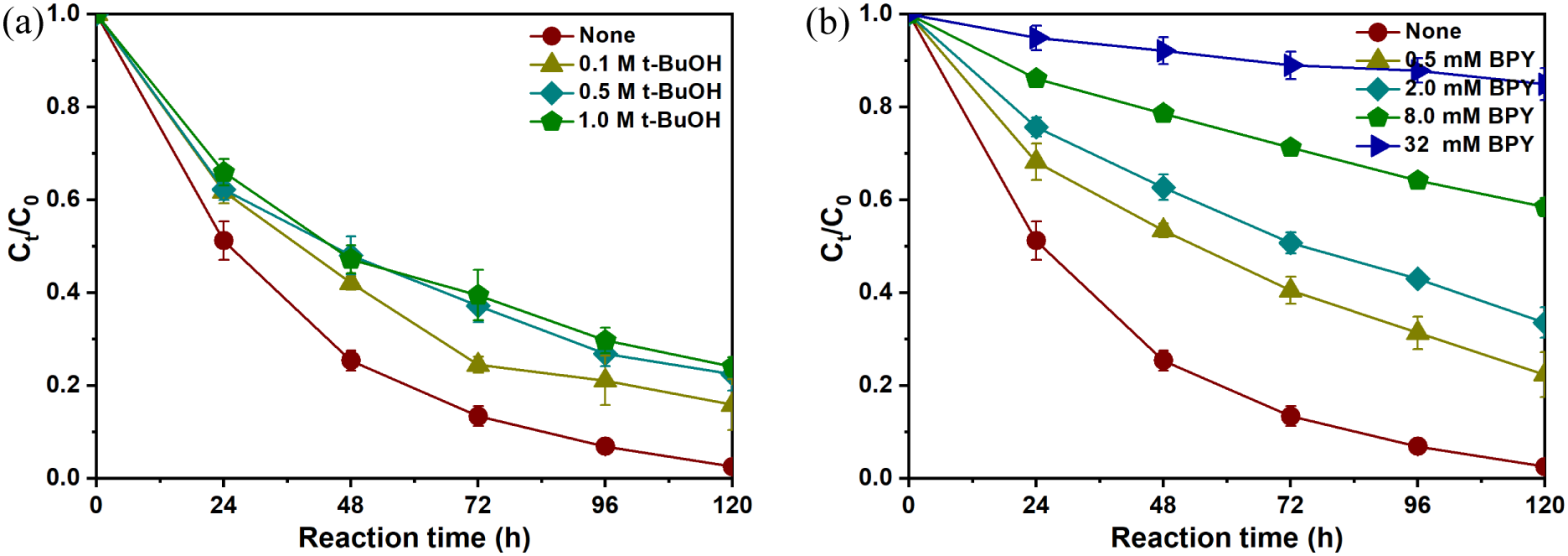
Effect of (a) t-BuOH and (b) BPY on NO_3_^–^ reduction in the FeS system. Experimental conditions: [FeS]_0_ = 200 g/L, [NO_3_^–^–N]_0_ = 30 mg/L and *T* = 30 °C, without pH adjustment.

To assess the role of •H in the NO_3_^–^ chemodenitrification on FeS, experiments were conducted with tertiary butanol (t-BuOH), a scavenger of •H that converts it into inert 2-methyl-2-propanol radicals.^43^ As depicted in Figure 7a, the efficiency of NO_3_^–^ chemodenitrification decreased from 97.5% to 84.2% with t-BuOH (0.1 M) and then stabilized with increasing t-BuOH dosage. This suggests that approximately 13% of NO_3_^–^ chemodenitrification might be attributed to surface-adsorbed •H. Consequently, it can be inferred that the majority of the NO_3_^–^ reduction was not mediated by reducing •H but by direct electron transfer coupled with H_ads_ formation facilitated by H^+^ on the FeS surface. To further explore the NO_3_^–^ chemodenitrification mechanism at the active sites on the FeS surface, 2,2’–bipyridyl (BPY), capable of chelating Fe(II) to impede electron transfer to the oxidants on the particle surface,^44^ was introduced into the reaction system. As anticipated, the chemodenitrification efficiency of NO_3_^–^–N by FeS decreased from 97.5% to 66.5% in the presence of BPY (2.0 mM) and then decreased to 15.1% as the BPY dosage was increased to 32 mM (Figure 7b), confirming that Fe(II) was the main active site on FeS for NO_3_^–^ chemodenitrification.

From the above results, high concentrations of sulfur vacancies existed in the FeS, and it provided more active sites for NO_3_^–^ reduction. The vacancies can also increase the adsorption capacity of FeS for NO_3_^–^, allowing for the more efficient reduction of NO_3_^–^ to NH_4_^+^. DFT calculations were further employed to examine the adsorption behavior of the NO_3_^–^ and H_2_O molecules on the sulfur vacancies on the FeS surface. Following the optimization of the most stable adsorption modes, the calculated adsorption energy (*E*_ads_) of O atoms in the NO_3_^–^ and H_2_O molecule on the sulfur vacancy of FeS surface was −1.96 eV and −0.82 eV (Table S3). This value was much lower than that of the N atom (−0.79 eV) in the NO_3_^–^ molecule and the H atom (−0.19 eV) in the H_2_O molecule on the FeS surface with sulfur vacancy available (Table S3), suggesting favorable adsorption of the O atoms of the NO_3_^–^ and H_2_O molecule onto the sulfur vacancy-rich FeS. Furthermore, the bond dissociation energy (D_0_) for the O atom and N atom within the NO_3_^–^ molecule on the sulfur vacancy of the FeS surface was determined to be 0.21 and 1.03 eV, respectively (Table S3), indicating that the other O atoms in the NO_3_^–^ were preferentially dissociated on the sulfur vacancies. Similarly, the D_0_ values of the O atom and the H atom in the H_2_O molecule on the sulfur vacancy of the FeS surface were 0.75 and 0.40 eV, respectively (Table S3), and implied that H atoms in H_2_O molecule were more prone to dissociation, forming H_ads_, which interacted with the deoxygenated NO_3_^–^. This sequence of deoxygenation and hydrogenation processes actively promoted the formation of NH_4_^+^.

**Figure 8:**
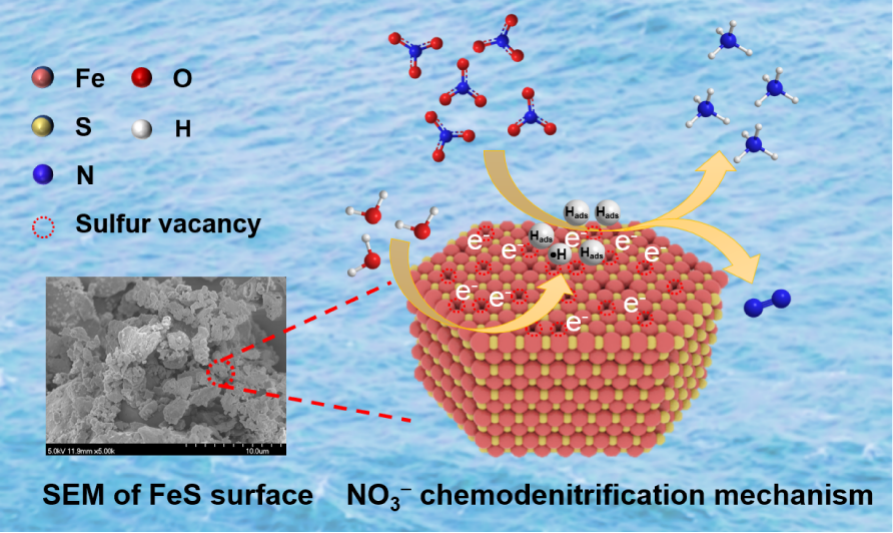
Schematic illustration of the NO_3_^–^ chemodenitrification mechanism in the FeS system.

Based on the above results and systematic discussion, the possible reduction pathways of the NO_3_^–^–FeS system is proposed and shown in Figure 8. Approximately 13% of the NO_3_^–^ reduction (Figure 7a) is related to the role of •H generated by sulfur vacancies on the FeS surface and H_2_O molecules. Most of the NO_3_^–^ reduction is dominated by direct electron transfer and H_ads_. In general, the O atoms of NO_3_^–^ tend to be adsorbed on the sulfur vacancy sites. At the same time, the O atoms in water molecules are adsorbed on the sulfur vacancies, followed by water dissociation, which generates H_ads_ on the nearby sulfur vacancy sites. Then, N-intermediates adsorbed on the sulfur vacancies of FeS are converted to NH_4_^+^ by electron transfer coupled with hydrogenation, and a small number of N-intermediates are converted to N_2_.

## Environmental Implications

Three kinds of iron sulfides, such as FeS, FeS_2_, and pyrrhotite, were studied for the NO_3_^–^ chemodenitrification under mild conditions. Chemical NO_3_^–^ reduction by FeS occurred but not by FeS_2_ and pyrrhotite. FeS had a high concentration of sulfur vacancies and quickly released electrons, which may be the main reason for its efficient chemical reduction of NO_3_^–^. Experimental results and DFT calculations demonstrate that Fe(II) on the FeS surface was the primary reactive site for NO_3_^–^ chemodenitrification and that FeS possessing sulfur vacancies exhibited the specific adsorption of O atoms within the NO_3_^–^ molecule and promoted intrinsic activity for H_ads_ formation through H_2_O dissociation, thus leading to a heightened selectivity in NH_4_^+^ formation (82.6%). Although NH_4_^+^ is considered an important indicator to nitrogen pollution in wastewater and water environment, NH_4_^+^/NH_3_ is a type of nitrogen fertilizer and even can be used as a fuel.^45,46^ Therefore, the highly selective NH_4_^+^ production by means of chemodenitrification of NO_3_^–^ allows us to envision that NO_3_^–^, a pollutant, might be recovered as a fertilizer and fuel through this chemodenitrification process. However, comprehensive research on real wastewater should be conducted in the future. In addition, this research helps in improving our understanding of the basic science behind chemical NO_3_^–^ reduction and, in turn, advancing the NO_3_^–^ remediation by iron sulfides-based technology. Furthermore, this work also provides broader insights into the field of geochemistry for understanding the complexity of N, Fe, and S cycles in ecosystems and even the conditions for the emergence of life on early Earth. However, extrapolating these results to a broader context requires careful consideration of the limitations inherent in laboratory experiments and the complexity of the Hadean Earth environment.

## Supporting Information Available

Analytical methods; density functional theory (DFT) calculations; variation of NO_2_^–^–N during the reduction of NO_3_^–^ by iron sulfides; effects of operation parameters on the ratios of NH_4_^+^ production vs NO_3_^–^ reduction for FeS; plot of ln k and 1/T of the NO_3_^–^ reduction; effects of different types of nitrate and cations on NO_3_^–^ removal and the ratios of NH_4_^+^ production vs NO_3_^–^ reduction by iron sulfides; SEM images, element components, and XPS results based on curve fitting for Fe 2p and S 2p peaks of FeS, FeS_2_, and pyrrhotite; variation of NO_3_^–^–N during the reduction of NO_3_^–^ by iron power, FeS, and a mixture of iron power and FeS; adsorption energies (*E*_ads_) and bond dissociation energy (D_0_) of the atoms in the H_2_O and NO_3_^–^ molecules on the sulfur vacancy on the FeS (114) surfaces (PDF)
