# Supplementary material for: Nitrate Chemodenitrification by Iron Sulfides to Ammonium under Mild Conditions and Transformation Mechanism
Source: Environ Sci Technol. 2024 May 21;58(22):9804–14. doi: 10.1021/acs.est.4c00195 (PMC11154956; doi:10.1021/acs.est.4c00195)
Supplement: Supplementary file 1 — es4c00195_si_001.pdf [file es4c00195_si_001.pdf]

Supporting Information (SI)

Nitrate Chemodenitrification by Iron Sulfides to Ammonium under Mild Conditions and Transformation Mechanism

Huanhuan Hu,[†] *Yang Bai,*[†] *Chong–Wen Zhou,*[‡, §] *Weihang Jia,*[§] *Piet N.L. Lens,*[‖] *Zhenhu Hu,*[⊥] *David Caffrey,*[#] *Xinmin Zhan*[†, *]

† Civil Engineering, School of Engineering, College of Science and Engineering, University of Galway, Galway H91 TK33, Ireland

‡ Combustion Chemistry Centre, School of Biological and Chemical Sciences, Ryan Institute, University of Galway, Galway H91 TK33, Ireland

§ School of Energy and Power Engineering, Beihang University, Beijing 100191, China

‖ Department of Microbiology, University of Galway, Galway H91 TK33, Ireland

⊥ Department of Municipal Engineering, School of Civil Engineering, Hefei University of Technology, Hefei 230009, China

School of Physics, Trinity College Dublin, Dublin 2, Ireland

Number of pages in supporting information: 15

Number of figures: 6

Number of tables: 3

Contents

Text S1. Analytical methods. S3

Text S2. Density functional theory (DFT) calculations. S5

Figure S1. Variation of NO_2^-−N during the reduction of NO_3^- by iron sulfides. S6

Figure S2. Effects of operation parameters on the ratios of NH_4^+ production vs NO_3^- reduction for FeS: (a) Mass ratio of FeS/N, (b) Temperature and (c) pH value. Except for the investigated parameters, other parameters were fixed: $[FeS]_0$ = 200 g/L, $[NO_3^-−N]_0$ = 30 mg/L, T = 30 °C, without pH adjustment. .. S7

Figure S3. Plot of ln k and 1/T of the NO_3^- reduction. S8

Figure S4. Effects of different types of nitrate and cations on (a) NO_3^- removal and (b) ratios of NH_4^+ production vs NO_3^- reduction by iron sulfides. Except for the investigated parameters, other parameters were fixed: [iron sulfide]$_0$ = 200 g/L, $[NO_3^-−N]_0$ = 30 mg/L, T = 30 °C, without pH adjustment. S8

Figure S5. SEM images of (a) FeS, (b) FeS_2, and (c) pyrrhotite samples. S8

Figure S6. Variation of NO_3^-−N during the reduction of NO_3^- by iron power, FeS, and a mixture of iron power and FeS. S9

Table S1. Element components of FeS, FeS_2 and pyrrhotite (mass ratio, %). S9

Table S2. XPS results based on curve fitting for Fe 2p and S 2p peaks of FeS, FeS_2 and pyrrhotite before and after the reaction shown in Figure 5. .... S9

Table S3. Adsorption energies (E_{ads}) and bond dissociation energy (D_0) of the atoms in the H_2O and NO_3^- molecules on the sulfur vacancy on the FeS (114) surfaces. S11

References S12

Text S1. Analytical methods.

The concentrations of NO_3^-–N, NH_4^+–N, NO_2^-–N, and SO_4^{2-} were determined using a nutrient analyzer (ThermoScientific, Gallery Plus, Waltham, United States) or ion chromatography (IC, ThermoScientific, Dionex Aquion, Waltham, United States). Because the high concentrations of FeS_2, pyrrhotite, Fe^{2+}, S^{2-}, HS^-, $S_2O_3^{2-}$ and SO_3^{2-} might affect the determination of NO_3^- by the nutrient analyzer, IC was used. The kinetics of NO_3^-–N chemodenitrification by the FeS system was modelled utilizing the apparent pseudo–first–order reaction rate model, expressed as eq S1:

$$C_t = C_0 \times e^{-kt} \quad \text{(S1)}$$

where, C_0 represents the initial concentration of NO_3^-–N (mg/L), C_t signifies the NO_3^-–N concentration at a specific time after the reaction initiation (mg/L), t denotes the reaction time (h), and k represents the apparent rate constant (h^{-1}).

According to the Arrhenius Equation, the plots of ln k and 1/T are expressed as eq S2:

$$ln\ k = -\frac{Ea}{RT} + ln\ A \quad \text{(S2)}$$

where Ea represents the activation energy (kJ/mol), T is the temperature (K), R is the molar gas constant (0.008314 kJ mol^{-1} K^{-1}), and A represents the pre−exponential factor (expressed in the same unit as k).

The total concentrations of Fe in the solution after reaction were measured using inductively coupled plasma mass spectrometry (ICP–MS, Agilent, 7700, Santa Clara, United States) and Fe^{2+} was measured by the nutrient analyzer. Monitoring of solution pH was conducted by a portable pH meter (WTW, pH 3210, Weilheim, Germany). The concentrations of NO, NO_2 and N_2O in the headspace gas were measured by a FTIR gas analyzer (Gasmet, DX–4000, Vantaa, Finland). First, the inside of the gas mixture tank needed to be pumped to a vacuum state, and then the syringe (internal vacuum) was insert into the serum glass bottle containing wastewater sealed with a nitrile rubber stopper. Due to the gas pressure inside the bottle being greater than atmospheric pressure, the gas inside the bottle automatically flew into the injector

(approximately 5 mL of gas each time), and the gas was filled into the gas distribution tank through the injection port, before diluted with nitrogen to 5 bar due to the gas chromatography detection of the sample. The sample gas was introduced to the detector at a flow rate of 5 L/min for 4 − 8 minutes, so a mixture with sufficient pressure needed to be configured.

Tafel scans were performed to determine the free corrosion potentials of the three iron sulfides on a three–electrode system (Autolab, PGSTAT204, Herisau, Switzerland) immersed in 30 mg/L NO_3^-–N solution at ambient temperature. The potential interval of −0.8 V to 0.8 V, with a 10 mV/s scan rate, was set to obtain the polarization curves. The counter electrode and reference electrode were a Pt wire and Ag/AgCl in 3 M KCl, respectively. The working electrode was made of 10.0 mg of pristine FeS, FeS_2 or pyrrhotite in 600 μL of deoxygenated ultrapure water, 300 μL of isopropanol, and 100 μL of a 5% Nafion solution.[1] X–ray fluorescence (XRF, Rigaku EDXRF, Tokyo, Japan) was used to analyze the elemental composition of FeS, FeS_2, and pyrrhotite that were not exposed to NO_3^-. The crystal structures of FeS, FeS_2, and pyrrhotite before and after the reaction were examined through X–ray diffraction (XRD) utilizing a Bruker D8 Discover (Billerica, United States) with a monochromatic Cu Kα source. To analyze the element transformation on the surface of FeS, FeS_2, and pyrrhotite, X–ray photoelectron spectroscopy (XPS, ThermoFischer, ESCALAB Xi+, Waltham, United States) with a monochromised Al Kα source (hv=1486.6 eV) was employed. Morphological observations were conducted using field emission scanning electron microscopy (FESEM, Hitachi, S–4700, Tokyo, Japan). For the XRD and XPS characterization of the iron sulfides after reaction, the used iron sulfides in the suspension were collected, subjected to thorough washing with deionized water, and subsequently dried in N_2 atmosphere at 80 ^oC. A Zetasizer equipment (Malvern Instruments, Nano ZS90, Worcestershire, United Kingdom) was used to measure the surface zeta–potential of FeS.

In this study, the distribution of sulfur species with changes in pH value was predicted using the Multi−problem/Sweep function in the Visual MINTEQ 3.1 model. First, the calculation of pH in the main menu was set to ‘Fixed at 2’, the calculation of the ionic strength was set to ‘To be calculated’, and the

temperature and concentration units were set to '30 °C' and 'Molal'. Also, sulfur species (S(−II), S(0), $S_2O_3^{2-}$, SO_3^{2-}, SO_4^{2-}) possibly present in this reaction system were input in 'Add components'. Second, running of the modelling was performed by setting 'Multi−problem/Sweep'. The sweep option was 'Sweep: one parameter is varied'. To simulate the distribution of sulfur species in the range of pH 2 − 12, the 'start value' and 'increment between values' were set to '2' and '0.1' respectively, the 'state the number of problems' was set to '100', and the sulfur species (HS^-, H_2S (aq), S^{2-}, SO_3^{2-}, HSO_3^-, SO_4^{2-}, and $S_2O_3^{2-}$) were selected for sweep output as pH 2 − 12.

Text S2. Density functional theory (DFT) calculations.

Spin–polarized DFT calculations were conducted using the Quantum Espresso (v.7.2)[2, 3] with ultrasoft (US) Vanderbilt–type pseudopotentials[4] and a plane–wave cutoff of 50 Ry. The general gradient approximation (GGA)[5] with the Perdew–Burke–Ernzerhof (PBE) parametrization was utilized for the exchange–correlation (XC) functional.[6, 7] Structural optimizations were performed using the quasi–Newton method, with convergence criteria set at 10^{-4} Ry for total energy and 10^{-3} Ry/Bohr for force. For numerical convergence, a 3×3×1 Monkhorst–Pack *k*–point grid was applied for FeS, and partial occupancies were determined by the Fermi–Dirac and Methfessel–Paxton method with a set width of 0.002 Ry for all calculations. To accurately represent van der Waals (vdw) interactions in the adsorption, the non–local van der Waals density functional (vdw–DF)[8-11] was incorporated. The FeS bulk structure is magnetic and spin–polarized calculations were used in all FeS calculations. The adsorption energy of the adsorbate (E_{ads}) was calculated by the adsorption energy eq S3:

$$E_{\text{ads}} = E_{\text{adsorbate+surface}} - E_{\text{adsorbate}} - E_{\text{surface}} \quad \text{(S3)}$$

where $E_{\text{adsorbate+surface}}$, $E_{\text{adsorbate}}$ and E_{surface} represent the total energies of the adsorbate–surface systems, the adsorbate species and the pristine surface, respectively.

Based on the XRD result, it was observed that FeS exhibited a hexagonal structure (space group P63/mmc) with lattice parameters a = b = 5.968 Å, c = 11.761 Å and c/a ratio = 1.971. Upon conducting

a full unit relaxation, the unit cell parameters were determined to be a = b = 5.949 Å, c = 11.721 Å and c/a ratio = 1.970. These values align well with the XRD results obtained in this study. In this calculation, the (114) facet for FeS was selected, which was mostly exposed as demonstrated by the XRD results. For the FeS (114) surface, which contains a four–layer slab, there are two layers fixed and all the other atoms are allowed to relax freely. To prevent interactions between periodic slabs, a vacuum spacing of 12 Å was incorporated in the z–direction. Meanwhile, a sulfur atom was removed to construct the sulfur vacancy on the surface of FeS (114).

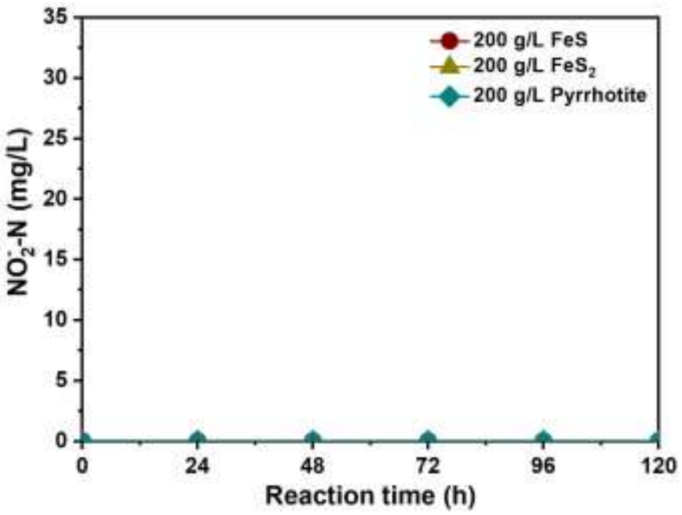

Figure S1. Variation of NO_2^-−N during the reduction of NO_3^- by iron sulfides.

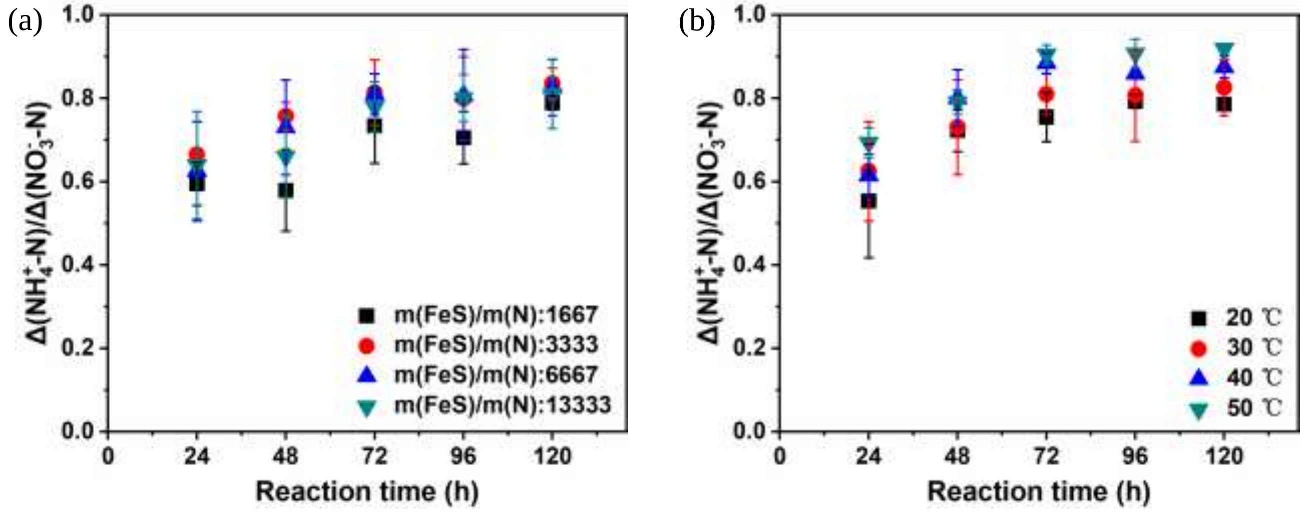

(c)

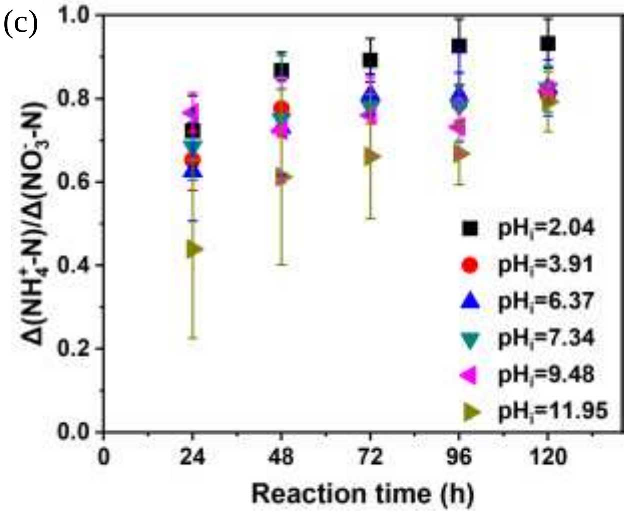

Figure S2. Effects of operation parameters on the ratios of NH_4^+ production vs NO_3^- reduction for FeS: (a) Mass ratio of FeS/N, (b) Temperature and (c) pH value. Except for the investigated parameters, other parameters were fixed: $[FeS]_0$ = 200 g/L, $[NO_3^-{-}N]_0$ = 30 mg/L, T = 30 °C, without pH adjustment.

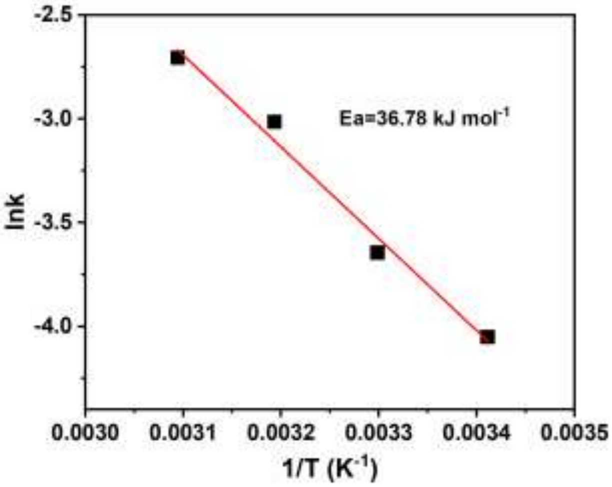

Figure S3. Plot of ln k and 1/T of the NO_3^- reduction.

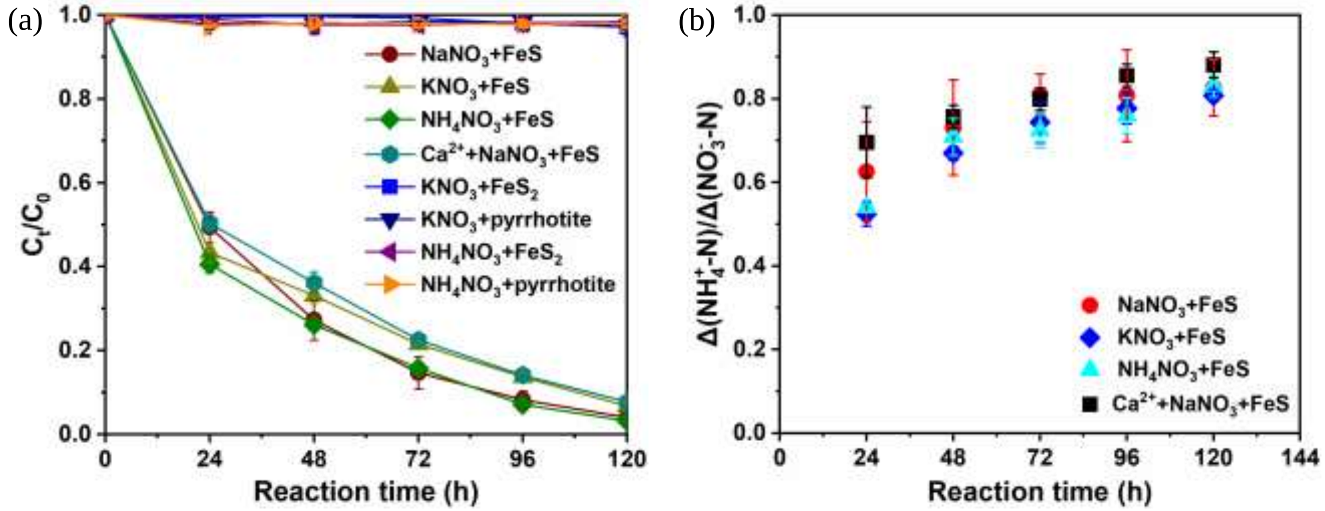

Figure S4. Effects of different types of nitrate and cations on (a) NO_3^- removal and (b) ratios of NH_4^+ production vs NO_3^- reduction by iron sulfides. Except for the investigated parameters, other parameters were fixed: [iron sulfide]$_0$ = 200 g/L, [NO_3^-−N]$_0$ = 30 mg/L, T = 30 °C, without pH adjustment.

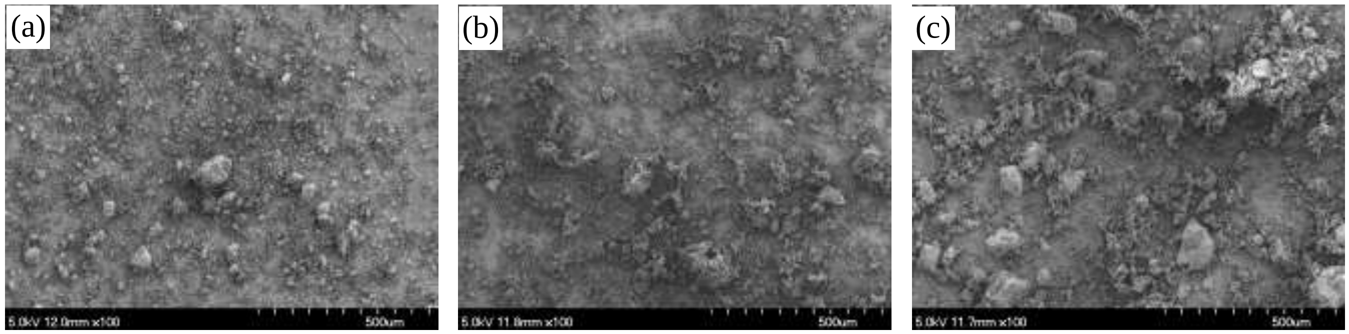

Figure S5. SEM images of (a) FeS, (b) FeS_2, and (c) pyrrhotite samples.

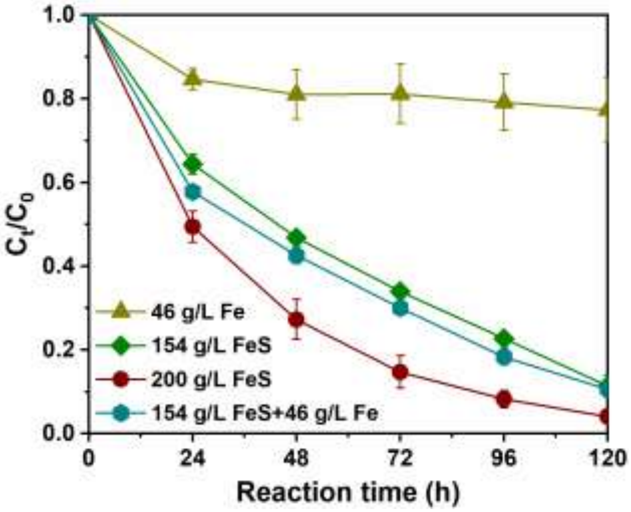

Figure S6. Variation of NO_3^-−N during the reduction of NO_3^- by iron power, FeS, and a mixture of iron power and FeS.

Table S1. Element components of FeS, FeS_2 and pyrrhotite (mass ratio, %).

Samples	S	Fe	Si	Ca	Mg	Al	K	Cu	Zn	Mn	Cr
FeS	27.60	71.50	0.28	–	–	0.26	–	0.019	0.006	0.23	0.045
FeS_2	39.90	45.40	6.02	2.04	3.04	2.48	0.11	0.44	0.096	0.24	0.23
Pyrrhotite	34.90	62.60	0.90	0.21	0.43	0.39	–	0.51	0.011	0.068	0.051

Table S2. XPS results based on curve fitting for Fe 2p and S 2p peaks of FeS, FeS_2 and pyrrhotite before and after the reaction shown in Figure 5.

Elements		Binding Energy (eV)	Species	Relative fraction (%)	References
Fe 2p	Pristine FeS	710.61	Fe(II)−S	14.87	12, 13
		712.16	Fe(III)−O	37.17	14
		718.20	Fe(III)−O	18.59	15
		724.08	Fe(II)−O	6.69	14, 15
		725.85	Fe(III)−O	16.36	14
		731.99	Fe(II)−O	6.32	15
	Used FeS	710.32	Fe(II)−S	8.06	12, 13
		711.44	Fe(III)−O	47.39	14
		719.29	Fe(III)−O	17.06	15
		723.72	Fe(II)−O	3.79	14, 15
		725.09	Fe(III)−O	20.86	14
		733.28	Fe(II)−O	2.84	15
S 2p	Pristine FeS	161.46	S(−II)	15.03	16
		162.59	S_2(−II)	16.43	17
		163.43	S_n(−II) (n=3, 4 …)	18.18	18
		168.61, 169.64	S(VI)	50.35	19

		161.46	S(−II)	7.84	[16]
	Used FeS	163.43	S_n(−II) (n=3, 4, …)	32.94	[18]
		168.57, 169.64	S(VI)	59.22	[19]
Fe 2p	Pristine FeS_2	707.24	Fe(II)−S	18.10	[20, 21]
		711.96	Fe(III)−O	45.25	[21]
		714.89	Fe(III)−O	11.31	[22]
		720.04	Fe(II)−S	4.52	[20, 21]
		725.85	Fe(III)−O	20.81	[20]
	Used FeS_2	707.07	Fe(II)−S	23.66	[20, 21]
		711.75	Fe(III)−O	53.76	[21]
		719.90	Fe(II)−S	5.38	[20, 21]
		725.64	Fe(III)−O	17.20	[20]
S 2p	Pristine FeS_2	162.68	S_2(−II)	19.41	[20, 23]
		163.88	S_n(−II) (n=3, 4, …)	17.11	[20]
		168.84, 169.94	S(VI)	63.49	[19]
	Used FeS_2	162.33	S_2(−II)	36.76	[20, 23]
		163.56	S_n(−II) (n=3, 4, …)	30.51	[20]
		168.84, 169.94	S(VI)	32.72	[19]
Fe 2p	Pristine pyrrhotite	707.31	Fe(II)−S	4.02	[24, 25]
		711.03	Fe(III)−O	22.89	[24, 25]
		712.93	Fe(II)−O	40.16	[26]
		720.09	Fe(II)−S	4.02	[20, 21]
		723.98	Fe(II)−O	10.44	[14, 15]
		725.57	Fe(III)−O	18.47	[20]
	Used pyrrhotite	707.23	Fe(II)−S	7.26	[24, 25]
		710.79	Fe(III)−O	25.42	[24, 25]
		712.44	Fe(II)−O	27.93	[26]
		719.91	Fe(II)−S	6.15	[20, 21]
		724.29	Fe(II)−O	15.36	[14]

		726.03	Fe(III)−O	17.88	[20]
S 2p	Pristine pyrrhotite	161.53	S(−II)	5.43	[26]
		162.98	S_2(−II)	28.96	[26]
		164.28	S_n(−II) (n=3, 4 …)	12.67	[26]
		166.92	S(IV)	1.81	[24, 27]
		168.93, 170.23	S(VI)	51.13	[26]
	Used pyrrhotite	161.46	S(−II)	16.17	[26]
		162.70	S_2(−II)	9.58	[26]
		163.80	S_n(−II) (n=3, 4 …)	29.94	[26]
		167.05	S(IV)	3.89	[24, 27]
		168.69, 169.90	S(VI)	40.42	[26]

Table S3. Adsorption energies (E_{ads}) and bond dissociation energy (D_0) of the atoms in the H_2O and NO_3^- molecules on the sulfur vacancy on the FeS (114) surfaces.

Species	H_2O−O	NO_3^-−O	H_2O−H	NO_3^-−N
E_{ads} (eV)	−0.82	−1.96	−0.19	−0.79
D_0 (eV)	0.75	0.21	0.40	1.03

References

1. Liu, Y.; Gan, H.; Tian, L.; Liu, Z.; Ji, Y.; Zhang, T.; Alvarez, P. J. J.; Chen, W., Partial oxidation of FeS nanoparticles enhances Cr(VI) sequestration. *Environmental Science & Technology* **2022,** *56* (19), 13954-13963, https://doi.org/10.1021/acs.est.2c02406.

2. Giannozzi, P.; Baroni, S.; Bonini, N.; Calandra, M.; Car, R.; Cavazzoni, C.; Ceresoli, D.; Chiarotti, G. L.; Cococcioni, M.; Dabo, I.; Dal Corso, A.; de Gironcoli, S.; Fabris, S.; Fratesi, G.; Gebauer, R.; Gerstmann, U.; Gougoussis, C.; Kokalj, A.; Lazzeri, M.; Martin-Samos, L.; Marzari, N.; Mauri, F.; Mazzarello, R.; Paolini, S.; Pasquarello, A.; Paulatto, L.; Sbraccia, C.; Scandolo, S.; Sclauzero, G.; Seitsonen, A. P.; Smogunov, A.; Umari, P.; Wentzcovitch, R. M., Quantum Espresso: A modular and open-source software project for quantum simulations of materials. *Journal of Physics: Condensed Matter* **2009,** *21* (39), 395502, https://doi.org/10.1088/0953-8984/21/39/395502.

3. Giannozzi, P.; Andreussi, O.; Brumme, T.; Bunau, O.; Buongiorno Nardelli, M.; Calandra, M.; Car, R.; Cavazzoni, C.; Ceresoli, D.; Cococcioni, M.; Colonna, N.; Carnimeo, I.; Dal Corso, A.; de Gironcoli, S.; Delugas, P.; DiStasio, R. A.; Ferretti, A.; Floris, A.; Fratesi, G.; Fugallo, G.; Gebauer, R.; Gerstmann, U.; Giustino, F.; Gorni, T.; Jia, J.; Kawamura, M.; Ko, H. Y.; Kokalj, A.; Küçükbenli, E.; Lazzeri, M.; Marsili, M.; Marzari, N.; Mauri, F.; Nguyen, N. L.; Nguyen, H. V.; Otero-de-la-Roza, A.; Paulatto, L.; Poncé, S.; Rocca, D.; Sabatini, R.; Santra, B.; Schlipf, M.; Seitsonen, A. P.; Smogunov, A.; Timrov, I.; Thonhauser, T.; Umari, P.; Vast, N.; Wu, X.; Baroni, S., Advanced capabilities for materials modelling with Quantum Espresso. *Journal of Physics: Condensed Matter* **2017,** *29* (46), 465901, https://doi.org/10.1088/1361-648X/aa8f79.

4. Garrity, K. F.; Bennett, J. W.; Rabe, K. M.; Vanderbilt, D., Pseudopotentials for high-throughput DFT calculations. *Computational Materials Science* **2014,** *81*, 446-452, https://doi.org/10.1016/j.commatsci.2013.08.053.

5. Tang, W.; Sanville, E.; Henkelman, G., A grid-based Bader analysis algorithm without lattice bias. *Journal of Physics: Condensed Matter* **2009,** *21* (8), 084204, https://doi.org/10.1088/0953-8984/21/8/084204.

6. Kresse, G.; Joubert, D., From ultrasoft pseudopotentials to the projector augmented-wave method. *Physical Review B* **1999,** *59* (3), 1758-1775, https://doi.org/10.1103/PhysRevB.59.1758.

7. Perdew, J. P.; Burke, K.; Ernzerhof, M., Generalized gradient approximation made simple. *Physical Review Letters* **1996,** *77* (18), 3865-3868, https://doi.org/10.1103/PhysRevLett.77.3865.

8. Thonhauser, T.; Zuluaga, S.; Arter, C. A.; Berland, K.; Schröder, E.; Hyldgaard, P., Spin signature of nonlocal correlation binding in metal-organic frameworks. *Physical Review Letters* **2015,** *115* (13), 136402, https://doi.org/10.1103/PhysRevLett.115.136402.

9. Thonhauser, T.; Cooper, V. R.; Li, S.; Puzder, A.; Hyldgaard, P.; Langreth, D. C., Van der Waals density functional: Self-consistent potential and the nature of the van der Waals bond. *Physical Review B* **2007,** *76* (12), 125112, https://doi.org/10.1103/PhysRevB.76.125112.

10. Berland, K.; Cooper, V. R.; Lee, K.; Schröder, E.; Thonhauser, T.; Hyldgaard, P.; Lundqvist, B. I., Van der Waals forces in density functional theory: A review of the vdW-DF method. *Reports on Progress in Physics* **2015,** *78* (6), 066501, https://doi.org/10.1088/0034-4885/78/6/066501.

11. Langreth, D. C.; Lundqvist, B. I.; Chakarova-Käck, S. D.; Cooper, V. R.; Dion, M.; Hyldgaard, P.; Kelkkanen, A.; Kleis, J.; Kong, L.; Li, S.; Moses, P. G.; Murray, E.; Puzder, A.; Rydberg, H.; Schröder, E.; Thonhauser, T., A density functional for sparse matter. *Journal of Physics: Condensed Matter* **2009,** *21* (8), 084203, https://doi.org/10.1088/0953-8984/21/8/084203.

12. Carver, J. C.; Schweitzer, G. K.; Carlson, T. A., Use of X‐ray photoelectron spectroscopy to study bonding in Cr, Mn, Fe, and Co compounds. *The Journal of Chemical Physics* **1972,** *57* (2), 973-982, https://doi.org/10.1063/1.1678348.

13. Wu, J.; Zeng, R. J., In situ preparation of stabilized iron sulfide nanoparticle-impregnated alginate composite for selenite remediation. *Environmental Science & Technology* **2018,** *52* (11), 6487-6496, https://doi.org/10.1021/acs.est.7b05861.

14. Lyu, H.; Tang, J.; Huang, Y.; Gai, L.; Zeng, E. Y.; Liber, K.; Gong, Y., Removal of hexavalent chromium from aqueous solutions by a novel biochar supported nanoscale iron sulfide composite. *Chemical Engineering Journal* **2017,** *322*, 516-524, https://doi.org/10.1016/j.cej.2017.04.058.

15. Hong, Q.; Liu, C.; Wang, Z.; Li, R.; Liang, X.; Wang, Y.; Zhang, Y.; Song, Z.; Xiao, Z.; Cui, T.; Heng, B.; Xu, B.; Qi, F.; Ikhlaq, A., Electron transfer enhancing Fe(II)/Fe(III) cycle by sulfur and biochar in magnetic FeS@biochar to active peroxymonosulfate for 2,4-dichlorophenoxyacetic acid degradation. *Chemical Engineering Journal* **2021,** *417*, 129238, https://doi.org/10.1016/j.cej.2021.129238.

16. Zhang, H.; Peng, L.; Chen, A.; Shang, C.; Lei, M.; He, K.; Luo, S.; Shao, J.; Zeng, Q., Chitosan-stabilized FeS magnetic composites for chromium removal: Characterization, performance, mechanism, and stability. *Carbohydrate Polymers* **2019,** *214*, 276-285, https://doi.org/10.1016/j.carbpol.2019.03.056.

17. Yang, Y.; Chen, T.; Morrison, L.; Gerrity, S.; Collins, G.; Porca, E.; Li, R.; Zhan, X., Nanostructured pyrrhotite supports autotrophic denitrification for simultaneous nitrogen and phosphorus removal from secondary effluents. *Chemical Engineering Journal* **2017,** *328*, 511-518, https://doi.org/10.1016/j.cej.2017.07.061.

18. Wu, J.; Zhao, J.; Hou, J.; Zeng, R. J.; Xing, B., Degradation of tetrabromobisphenol A by sulfidated nanoscale zerovalent iron in a dynamic two-step anoxic/oxic process. *Environmental Science & Technology* **2019,** *53* (14), 8105-8114, https://doi.org/10.1021/acs.est.8b06834.

19. Cantrell, K. J.; Yabusaki, S. B.; Engelhard, M. H.; Mitroshkov, A. V.; Thornton, E. C., Oxidation of H_2S by iron oxides in unsaturated conditions. *Environmental Science & Technology* **2003,** *37* (10), 2192-2199, https://doi.org/10.1021/es020994o.

20. Ye, Z.; Padilla, J. A.; Xuriguera, E.; Beltran, J. L.; Alcaide, F.; Brillas, E.; Sirés, I., A highly stable metal–organic framework-engineered FeS_2/C nanocatalyst for heterogeneous electro-fenton treatment: Validation in wastewater at mild pH. *Environmental Science & Technology* **2020,** *54* (7), 4664-4674, https://doi.org/10.1021/acs.est.9b07604.

21. Lu, Z.; Wang, N.; Zhang, Y.; Xue, P.; Guo, M.; Tang, B.; Bai, Z.; Dou, S., Pyrite FeS_2@C nanorods as smart cathode for sodium ion battery with ultra-long lifespan and notable rate performance from tunable pseudocapacitance. *Electrochimica Acta* **2018,** *260*, 755-761, https://doi.org/10.1016/j.electacta.2017.12.031.

22. Guo, X.; Jia, J.; Xu, Y.; Meng, Q.; Zha, F.; Tang, X.; Tian, H., FeS_2-$Fe_{1-x}S$ heterostructure as a high-efficient Fenton-like catalyst for ultrafast degradation of orange II. *Applied Surface Science* **2021,** *556*, 149786, https://doi.org/10.1016/j.apsusc.2021.149786.

23. Zhang, P.; Huang, W.; Ji, Z.; Zhou, C.; Yuan, S., Mechanisms of hydroxyl radicals production from pyrite oxidation by hydrogen peroxide: Surface versus aqueous reactions. *Geochimica et Cosmochimica Acta* **2018,** *238*, 394-410, https://doi.org/10.1016/j.gca.2018.07.018.

24. Liu, J.; Li, E.; Jiang, K.; Li, Y.; Han, Y., Effect of acidic activators on the flotation of oxidized pyrrhotite. *Minerals Engineering* **2018,** *120*, 75-79, https://doi.org/10.1016/j.mineng.2018.02.017.

25. Liao, Y.; Chen, D.; Zou, S.; Xiong, S.; Xiao, X.; Dang, H.; Chen, T.; Yang, S., Recyclable naturally derived magnetic pyrrhotite for elemental mercury recovery from flue gas. *Environmental Science & Technology* **2016,** *50* (19), 10562-10569, https://doi.org/10.1021/acs.est.6b03288.

26. Yuan, Q.; Mei, G.; Liu, C.; Cheng, Q.; Yang, S., A novel sulfur-containing ionic liquid collector for the reverse flotation separation of pyrrhotite from magnetite. *Separation and Purification Technology* **2022,** *303*, 122189, https://doi.org/10.1016/j.seppur.2022.122189.

27. Qi, C.; Liu, J.; Malainey, J.; Kormos, L. J.; Coffin, J.; Deredin, C.; Liu, Q.; Fragomeni, D., The role of Cu ion activation and surface oxidation for polymorphic pyrrhotite flotation performance in Strathcona Mill. *Minerals Engineering* **2019,** *134*, 87-96, https://doi.org/10.1016/j.mineng.2019.01.025.
